# The role of Toll-like receptor-4 in pertussis vaccine-induced immunity

**DOI:** 10.1186/1471-2172-9-21

**Published:** 2008-05-22

**Authors:** Sander Banus, Rachel M Stenger, Eric R Gremmer, Jan AMA Dormans, Frits R Mooi, Tjeerd G Kimman, Rob J Vandebriel

**Affiliations:** 1 Laboratories for Infectious Diseases and Screening, National Institute of Public Health and the Environment, 3720 BA Bilthoven, The Netherlands; 2 Health Protection Research, National Institute of Public Health and the Environment, 3720 BA Bilthoven, The Netherlands; 3 Netherlands Vaccine Institute, 3720 AL Bilthoven, The Netherlands

## Abstract

**Background:**

The gram-negative bacterium *Bordetella pertussis *is an important causative agent of pertussis, an infectious disease of the respiratory tract. After introduction of whole-cell vaccines (wP) in the 1950's, pertussis incidence has decreased significantly. Because wP were found to be reactogenic, in most developed countries they have been replaced by acellular vaccines (aP). We have previously shown a role for Toll-like receptor 4 (Tlr4) in pertussis-infected mice and the pertussis toxin (Ptx)-IgG response in wP-vaccinated children, raising the issue of the relative importance of Tlr4 in wP vaccination of mice. Here we analyze the effects of wP and aP vaccination and *B. pertussis *challenge, in *Tlr4*-deficient C3H/HeJ and wild-type C3H/HeOuJ mice. aP consists of Ptx, filamentous hemagglutinin (FHA), and pertactin (Prn).

**Results:**

We show an important role of Tlr4 in wP and (to a lesser extent) aP vaccination, induction of Th1 and Th17 cells by wP but not aP vaccination, and induction of Th17 cells by infection, confirming data by Higgins et al. (*J Immunol *2006, **177:**7980–9). Furthermore, in *Tlr4*-deficient mice, compared to wild-type controls (i) after vaccination only, Ptx-IgG (that was induced by aP but not wP vaccination), FHA-IgG, and Prn-IgG levels were similar, (ii) after infection (only), lung IL-1α and IL-1β expression were lower, (iii) after wP vaccination and challenge, Prn-IgG level and lung IL-5 expression were higher, while lung IL-1β, TNF-α, IFN-γ, IL-17, and IL-23 expression were lower, and lung pathology was absent, and (iv) after aP vaccination and challenge, Prn-IgG level and lung IL-5 expression were higher, while Ptx-IgG level was lower.

**Conclusion:**

Tlr4 does not influence the humoral response to vaccination (without challenge), plays an important role in natural immunity, wP and aP efficacy, and induction of Th1 and Th17 responses, is critical for lung pathology and enhances pro-inflammatory cytokine production after wP vaccination and challenge, and diminishes Th2 responses after both wP and aP vaccination and challenge. wP vaccination does not induce Ptx-IgG. A role for LPS in the efficacy of wP underlines the usefulness of LPS analogs to improve bacterial subunit vaccines such as aP.

## Background

*Bordetella pertussis *is an important causative agent of pertussis, which is among the ten infectious diseases with the highest morbidity and mortality worldwide. After introduction of whole-cell vaccines (wP) in the 1950's, pertussis incidence has decreased significantly. Although efficacious, wP vaccines were also found to be reactogenic. Therefore, acellular vaccines (aP) comprising purified *B. pertussis *proteins have been developed.

Toll-like receptor 4 (Tlr4) is the receptor for lipopolysaccharide (LPS) in mammals [1]. Since *B. pertussis *is a gram-negative bacterium, Tlr4 is likely to play an important role in natural immunity to *B. pertussis *infection. Indeed, we and others have shown that Tlr4 is critical for *B. pertussis *clearance and ensuing adaptive immunity in non-vaccinated mice [2-4]. Furthermore, Tlr4 influenced lung pathology and production of proinflammatory cytokines, such as IL-1β and TNF-α, after *B. pertussis *infection [4].

Since wP are prepared from *B. pertussis *and (thus) contain LPS it may be suggested that Tlr4 influences wP efficacy. In our model of wP and aP vaccination and *B. pertussis *challenge, lung pathology and TNF-α expression were induced by vaccination (in particular with wP) and challenge [5]. Also, type I hypersensitivity, a Th2 driven response, was induced by vaccination (with both wP and aP) and challenge [5]. In that study, however, a role for Tlr4 was not addressed.

We have identified an association of the minor allele of rs2770150 in TLR4 with a lower pertussis toxin (Ptx)-IgG level in wP-vaccinated children [6]. The mouse and human studies together prompted us to analyze the relative role of Tlr4 in wP vaccination of mice.

Here we show that Tlr4 plays an important role in natural immunity, wP and (to a lesser extent) aP efficacy, and induction of Th1 and Th17 responses, confirming observations by Higgins et al. [7]. Moreover, we show that the humoral response to vaccination (without challenge) is not influenced by Tlr4 (wP vaccination did not induce detectable Ptx-IgG). Tlr4 is critical for lung pathology and enhances pro-inflammatory cytokine production after wP vaccination and challenge, and diminishes Th2 responses after both wP and aP vaccination and challenge.

## Results

### *B. pertussis *colonization of the lungs

**Figure 1:**
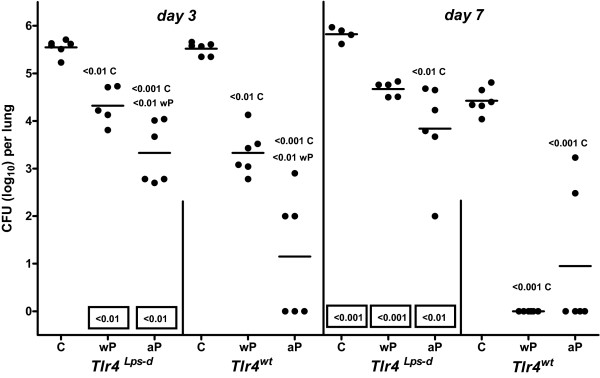
**Colonization of the lungs by *B. pertussis***. *Tlr4*-deficient C3H/HeJ and control C3H/HeOuJ mice were subcutaneously (sc) injected with 1/5 human dose (HD) wP, aP, or adjuvant (C), twice before intranasal *B. pertussis *infection. Three and seven days after challenge lungs were excised, and the number of viable *B. pertussis *was determined in right lung lobes. Each symbol represents the number of bacteria in the lung of an individual mouse; horizontal lines represent the group average. Non-boxed *P*-values: compared to the indicated treatment group (same strain and day after infection). Boxed *P*-values: compared to the wild-type strain (same treatment and day after infection). ANOVA followed by Bonferroni post-hoc test. A single representative experiment of 2 is shown.

*Tlr4*-deficient C3H/HeJ and wild-type control C3H/HeOuJ mice (6 per group) were vaccinated twice with 1/5 human dose (HD) wP, 1/5 HD aP, or adjuvant, and challenged intranasally with *B. pertussis*. Three and seven days after infection, mice were sacrificed and the number of bacteria in their lungs was determined. Vaccinated mice generally showed a lower number of bacteria than adjuvant-treated animals, while *Tlr4*-deficient mice generally showed a higher number of bacteria than wild-type animals (Figure 1). Adjuvant-treated *Tlr4*-deficient mice showed an increasing number of bacteria from day 3 till day 7 (Δ log CFU = 0.28, *P *= 0.029), while similarly treated wild-type animals showed a decreasing number in this time period (Δ log CFU = -1.09, *P *< 0.001). *Tlr4*-deficient mice that were wP-vaccinated before challenge failed to show a decreasing number of bacteria from day 3 till day 7, while similarly treated wild-type animals did show a decreasing number in this time period (Δ log CFU = -3.33, *P *< 0.001). In aP-vaccinated mice of either strain the number of bacteria was similar between day 3 and day 7.

These data show (i) the important role of Tlr4 in the natural defense to *B. pertussis *infection and confirms our earlier findings [4], (ii) the important role of Tlr4 in wP-induced clearance, and (iii) that vaccine-induced clearance is faster after aP vaccination than after wP vaccination, regardless of the presence of Tlr4. This latter notion is supported by the higher clearance in aP-vaccinated than wP-vaccinated mice at day 3 but not day 7 in either mouse strain.

Vaccination-induced protection, i.e. the difference in the number of bacteria between vaccinated and control mice, was lower in *Tlr4*-deficient mice than in wild-type animals (*P *< 0.01), except for aP vaccination at day 7 where this effect was not statistically significant (Δ log CFU = -1.23 and -2.19 (day 3, wP), -2.22 and -4.37 (day 3, aP), -1.15 and -4.43 (day 7, wP), and -1.99 and -3.48 (day 7, aP) for *Tlr4*-deficient and wild-type mice, respectively).

Collectively, these data show that Tlr4 is not only involved in the natural defense to *B. pertussis *infection but also in vaccination-induced protection to this pathogen, both after wP and after aP vaccination.

### Ptx-, FHA-, and Prn-specific IgG

**Figure 2:**
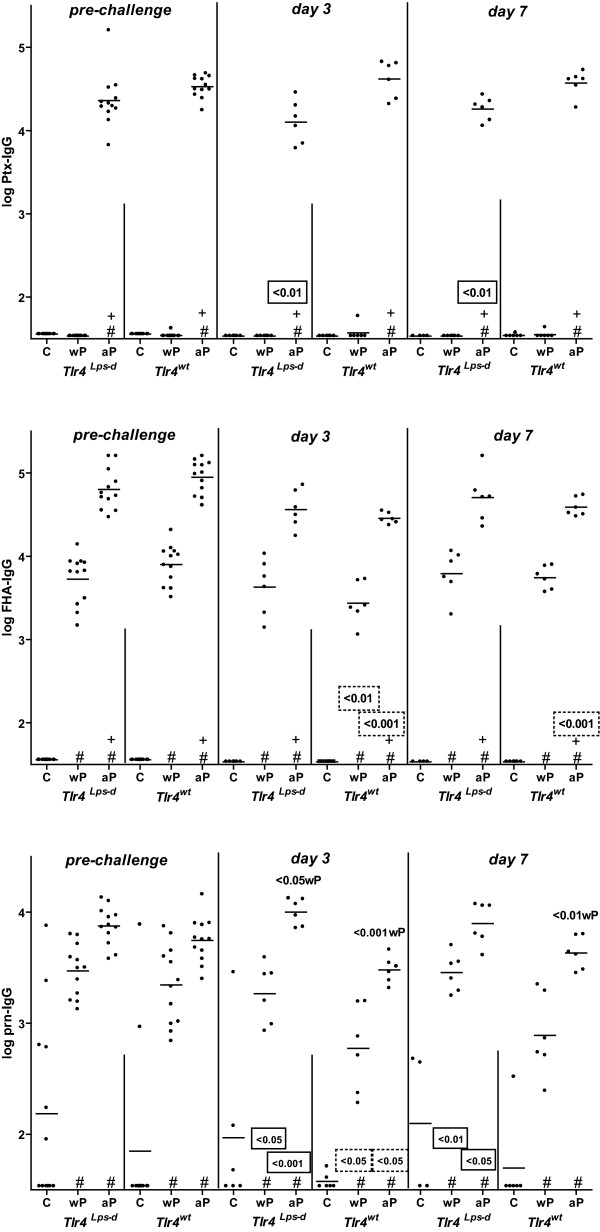
**Ptx-, FHA-, and Prn-specific IgG in serum**. *Tlr4*-deficient and control mice were sc injected with 1/5 HD wP, aP, or adjuvant (C), twice before intranasal *B. pertussis *infection. Two hours before challenge, and three and seven days after challenge blood was taken. Test and positive control sera were tested for Ptx-, FHA-, and Prn-specific IgG. Each symbol represents the serum level of an individual mouse; horizontal lines represent the group average. (#) increased compared to the adjuvant control (same strain and time point; *P *< 0.001), (+) increased compared to wP-vaccinated mice (same strain and time point; *P *< 0.001). Non-boxed *P*-values: compared to the indicated group (same strain and time point), boxed *P*-values: compared to the wild-type strain (same treatment and time point), and stippled-boxed P-values: compared to the pre-challenge level (same strain and treatment). ANOVA followed by Bonferroni post-hoc test. A single representative experiment of 2 is shown.

Serum was taken 2 hours before challenge and 3 and 7 days after challenge, and analyzed for IgG specific for Pertussis toxin (Ptx), filamentous hemagglutinin (FHA), and Pertactin 1 (Prn). Ptx-IgG was detectable only in aP-vaccinated mice (Figure 2). Before challenge the Ptx-IgG level was not affected by Tlr4. At days 3 and 7 after challenge *Tlr4*-deficient aP-vaccinated mice showed a significantly lower Ptx-IgG level than similarly treated wild-type animals. In each mouse strain the Ptx-IgG level was similar before and after challenge.

FHA-IgG was detectable in both wP- and aP-vaccinated mice. aP-vaccinated mice showed a higher FHA-IgG level than wP-vaccinated animals (irrespective of challenge). Both before and after challenge the FHA-IgG level was not affected by Tlr4. Generally, both wP- and aP-vaccinated wild-type but not *Tlr4*-deficient mice showed a lower FHA-IgG level after challenge than before.

Prn-IgG was detectable in both wP- and aP-vaccinated mice. Generally, aP-vaccinated mice showed a higher Prn-IgG level than wP-vaccinated animals. Before challenge the Prn-IgG level was not affected by Tlr4. Rather unexpectedly after challenge the Prn-IgG level was higher in wP- and aP-vaccinated *Tlr4*-deficient mice than in similarly treated wild-type animals.

Collectively, these data show that before challenge Tlr4 does not affect vaccination-induced Ptx-, FHA-, and Prn-IgG levels. After challenge, the Ptx-IgG level is lower in *Tlr4*-deficient mice than in wild-type animals, while the reverse is true for Prn-IgG. The FHA-IgG level is unaffected by the *Tlr4 *mutation.

### Weight gain

**Figure 3:**
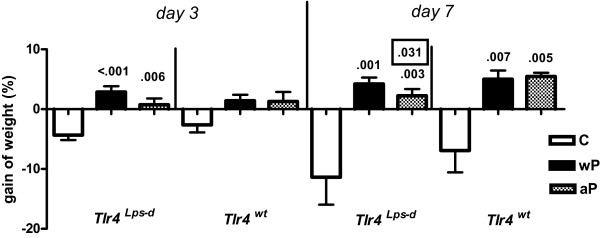
**Gain of weight**. *Tlr4*-deficient and control mice were sc injected with 1/5 HD wP, aP, or adjuvant (C), twice before intranasal *B. pertussis *infection. Two hours before challenge, and three and seven days after challenge mice were weighed. Data are indicated as mean ± SEM (N = 6). Non-boxed *P*-values: compared to the adjuvant control (same strain and day after challenge). Boxed *P*-value: compared to the wild-type strain (same treatment and day after challenge). ANOVA followed by Bonferroni post-hoc test. A single representative experiment of 2 is shown.

Mice were weighed immediately before challenge, and 3 and 7 days after challenge (Figure 3). Paired sample testing revealed weight loss of adjuvant controls; this was significant in case of *Tlr4*-deficient mice at day 3 (*P *< .003) and a trend in other cases. Weight gain was seen in wP- but not aP-vaccinated *Tlr4*-deficient mice at days 3 and 7, and in wP- and aP-vaccinated wild-type animals at day 7.

Weight gain in wP- and aP-vaccinated *Tlr4*-deficient mice was higher than in adjuvant controls at days 3 and 7, while in wP- and aP-vaccinated wild-type animals this was the case only at day 7. A lower weight gain due to the *Tlr4 *mutation was seen only in aP-vaccinated mice at day 7.

Taken together, these data show that vaccination but not (or to a much lesser extent) Tlr4 affects weight gain during the first 7 days after *B. pertussis *challenge.

### Histological changes

We have previously shown that after challenge wP- and aP-vaccinated mice revealed increased lung pathology compared to adjuvant-treated animals, with wP-vaccinated mice showing more severe pathology than aP-vaccinated animals [5]. Here we addressed whether Tlr4 influenced lung pathology.

**Figure 4:**
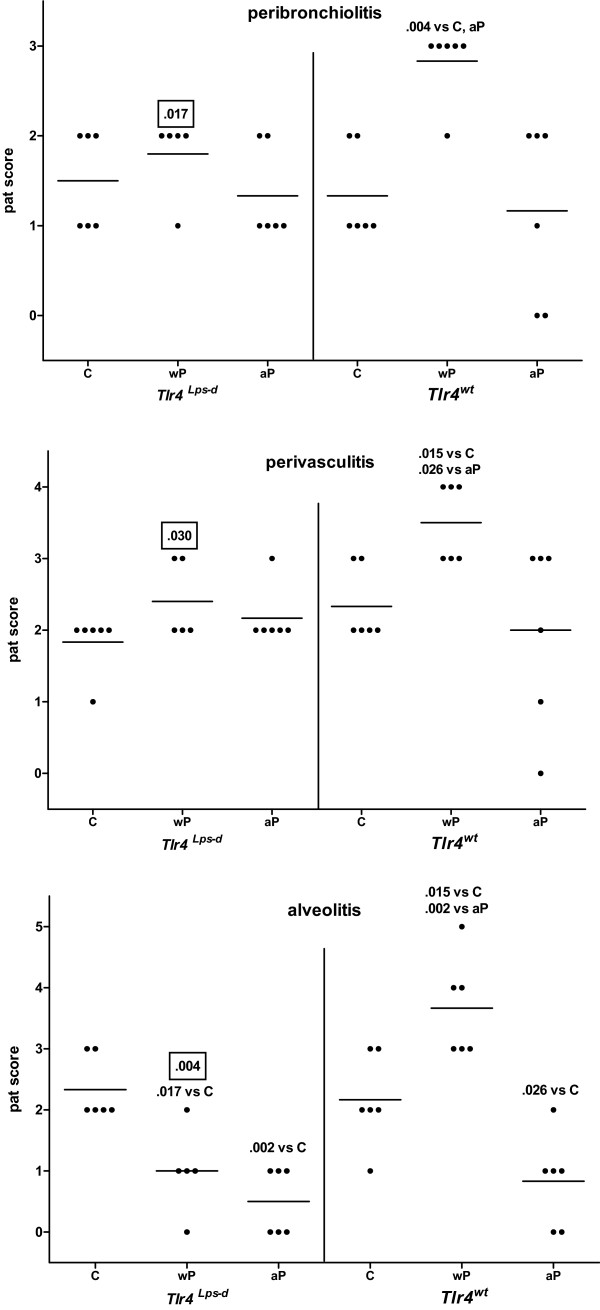
**Lung pathology**. *Tlr4*-deficient and control mice were sc injected with 1/5 HD wP, aP, or adjuvant (C), twice before intranasal *B. pertussis *infection. Three days after infection the left lung lobes were excised. H&E staining. Histological lesions were semi-quantitatively scored as absent (0), minimal (1), slight (2), moderate (3), strong (4), or severe (5), respectively. Each symbol represents an individual mouse; horizontal lines represent the group average. Non-boxed *P*-values: compared to the indicated group (same strain). Boxed *P*-values: compared to the wild-type strain (same treatment). Mann-Whitney U test. A single representative experiment of 2 is shown.

Lung lesions were scored 3 days after challenge (Figure 4). In wild-type mice wP vaccination resulted in a stronger challenge-induced peribronchiolitis, perivasculitis, and alveolitis compared to adjuvant treatment and aP vaccination, rather similar to our previous findings [5]. Challenge-induced alveolitis was weaker in wP- and aP-vaccinated *Tlr4*-deficient mice and in aP-vaccinated wild-type animals, compared to their adjuvant controls. No treatment effects on hypertrophy of the bronchiolar mucous cells were seen in either strain, while eosinophilia was absent in all groups.

Challenge-induced lung pathology was less severe in *Tlr4*-deficient wP-vaccinated mice than in similarly treated wild-type animals. No effect of the *Tlr4 *mutation was seen after challenge of aP-vaccinated and adjuvant-treated mice.

These data show that challenge-induced pathology in wP-vaccinated animals is Tlr4-dependent.

### Cytokine gene expression in lung tissue

*B. pertussis *infection induced TNF-α, IL-6, and IL-1β production and Tlr4 was shown to play a role in this process [2-4]. Thus, proinflammatory cytokines were analyzed. Next, wP-vaccination induced Th1 and Th17 cells, these cells were critical for clearance and their induction was shown to be dependent on Tlr4 [7]. Thus, Th1 and Th17 cytokines were analyzed. Finally, pertussis vaccination (in particular aP vaccination) before challenge induced a Th2-dependent response [5] and this response could be skewed towards a Th1 response by adding LPS analogs [8]. Thus, Th2 cytokines were analyzed.

Post-challenge lung lobes were subjected to real-time PCR. The fold change is relative to lung tissue of untreated C57BL/6J mice. Irrespective of prior vaccination, IL-1α mRNA expression is lower in challenged C3H mice than in untreated C57BL/6 mice, while the reverse is true for the other cytokines analyzed.

#### Proinflammatory cytokines

**Figure 5:**
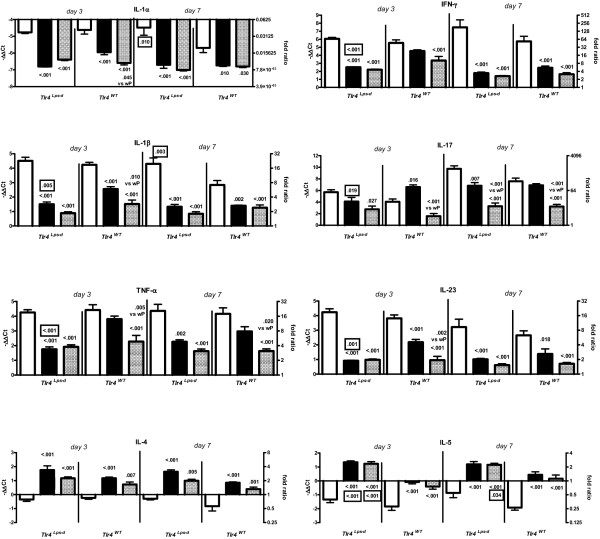
**Cytokine expression in lung**. *Tlr4*-deficient and control mice were sc injected with 1/5 HD wP (|▮|), aP (|▒|), or adjuvant (| |), twice before intranasal *B. pertussis *infection. Three and seven days after challenge the left lung lobes were excised. RNA was extracted and analyzed by real-time PCR. The fold change in mRNA expression is relative to lung tissue of untreated C57BL/6J mice. Data are indicated as mean ± SEM (N = 6). Non-boxed *P*-values: compared to the adjuvant control, or (when indicated) wP-vaccinated group (same strain and day after challenge). Boxed *P*-values: compared to wild-type mice (same treatment and day after challenge). ANOVA followed by Bonferroni. A single representative experiment of 2 is shown.

Generally, in both strains of mice wP and aP vaccination resulted in lower challenge-induced IL-1α, IL-1β, and TNF-α expression than adjuvant treatment (Figure 5). *Tlr4*-deficient adjuvant-treated mice showed higher challenge-induced IL-1α and IL-1β expression than similarly treated wild-type animals at day 7. *Tlr4*-deficient wP-vaccinated mice showed lower challenge-induced IL-1β and TNF-α expression than similarly treated wild-type animals at day 3.

#### Th1/Th17 cytokines

Generally, in both strains of mice wP and aP vaccination resulted in lower challenge-induced IFN-γ and IL-23 expression than adjuvant treatment, while for aP vaccination this was also seen for IL-17. Adjuvant-treated and aP-vaccinated mice did not show Tlr4-dependent effects on post-challenge Th1/Th17 cytokines. *Tlr4*-deficient wP-vaccinated mice did, however, show lower challenge-induced IFN-γ, IL-17, and IL-23 expression than similarly treated wild-type animals at day 3.

#### Th2 cytokines

Generally, in both strains of mice wP and aP vaccination resulted in higher challenge-induced IL-4 and IL-5 expression than adjuvant treatment. Adjuvant-treated mice did not show Tlr4-dependent effects on post-challenge Th2 cytokines. *Tlr4*-deficient aP-vaccinated mice showed higher challenge-induced IL-5 expression than similarly treated wild-type animals. This effect of the *Tlr4 *mutation was also seen for wP-vaccinated mice at day 3.

In summary, in both strains of mice wP and aP vaccination resulted in lower challenge-induced IL-1α, IL-1β, TNF-α, IFN-γ, IL-17, and IL-23 expression, and higher challenge-induced IL-4 and IL-5 expression. Exceptions to this overall picture were higher TNF-α and IL-17 expression in wP-vaccinated wild-type mice.

Tlr4 mediated (i) in adjuvant-treated mice, lower expression of IL-1α and IL-1β at day 7, (ii) in wP-vaccinated mice, higher expression of IL-1β and TNF-α, and of Th1 and Th17 cytokines at day 3, and (iii) in wP- and aP-vaccinated mice, higher expression of IL-5 at days 3 and 7 after challenge.

In short, functional Tlr4 results in a lower lung inflammatory response after infection, a higher inflammatory response and Th1/Th17 response after wP vaccination and challenge, and a lower Th2 response after wP and aP vaccination and challenge.

### Cytokine production by bronchial lymph node cells

Cell suspensions from post-challenge bronchial LN were Con A-stimulated *ex vivo*. From the panel of cytokines tested (IL-1α, IL-4, IL-5, IL-10, IL-13, IL-17, IFN-γ, and TNF-α), IL-1α and IL-13 were excluded from further analysis because for these cytokines one or more of the treatment groups showed concentrations below the detection limit for 4 or more of the 6 samples tested.

**Figure 6:**
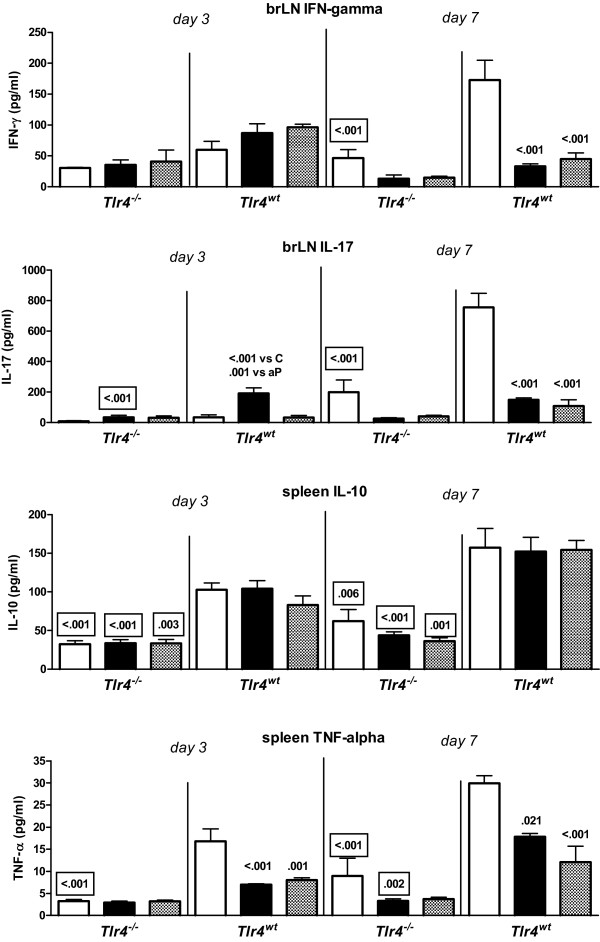
***Ex vivo *cytokine production by bronchial lymph node and spleen cells**. *Tlr4*-deficient and control mice were sc injected with 1/5 HD wP (|▮|), aP (|▒|), or adjuvant (| |), twice before intranasal *B. pertussis *infection. Three and seven days after challenge the bronchial lymph nodes (LN) and spleens were excised and cell suspensions were made. Bronchial LN cells were cultured with Con A for 24 hr; spleen cells were cultured with heat-killed *B. pertussis *for 72 hr. Culture supernatants were analyzed for cytokine content by Luminex. Data are indicated as mean ± SEM (N = 6). Non-boxed *P*-values: compared to the adjuvant control, or (when indicated) aP-vaccinated group (same strain and day after challenge). Boxed *P*-values: compared to wild-type mice (same treatment and day after challenge). ANOVA followed by Bonferroni. A single representative experiment of 2 is shown.

Generally, vaccinated mice showed lower challenge-induced IFN-γ and IL-17 production than adjuvant-treated animals at day 7 (Figure 6). *Tlr4*-deficient adjuvant-treated mice showed lower challenge-induced IFN-γ and IL-17 production than similarly treated wild-type animals at day 7. *Tlr4*-deficient wP-vaccinated mice showed lower challenge-induced IL-17 production than similarly treated wild-type mice at day 3.

IL-4, IL-5, and IL-10 production was 13-, 11-, and 9-fold higher, respectively, in wP-vaccinated than in adjuvant-treated *Tlr4*-deficient mice at day 3 after challenge (*P *= 0.004, *P *= 0.028, and *P *= 0.015, respectively). No strain- or additional treatment-related effects on the production of these cytokines were seen. No strain- or treatment-related effects on TNF-α production were seen (data not shown).

Together, in bronchial LN cells functional Tlr4 results in a higher Th1/Th17 response after infection. Moreover, in these cells functional Tlr4 results in a higher Th17 and a lower Th2 response after wP vaccination and challenge.

### Cytokine production by splenocytes

Cell suspensions from post-challenge spleens were stimulated *ex vivo *with heat-killed *B. pertussis*. From the panel of cytokines tested (IL-1α, IL-4, IL-5, IL-10, IL-13, IL-17, IFN-γ, and TNF-α), IL-1α, IL-4, IL-5, IL-13, IL-17, and IFN-γ were excluded from further analysis because for these cytokines one or more of the treatment groups showed concentrations below the detection limit for 4 or more of the 6 samples tested.

Vaccination did not affect challenge-induced IL-10 production, while *Tlr4*-deficient mice showed lower challenge-induced production of this cytokine than wild-type animals. *Tlr4*-deficient adjuvant-treated mice showed lower challenge-induced TNF-α production than similarly treated wild-type animals at days 3 and 7, while *Tlr4*-deficient wP-vaccinated mice showed lower challenge-induced production of this cytokine than similarly treated wild-type animals at day 7.

Together, these data show that in the spleen regardless of vaccination functional Tlr4 results in a higher IL-10 response after infection. Functional Tlr4 also results in a higher TNF-α response after infection, and after wP vaccination and challenge.

## Discussion

Here we have shown that after infection of non-vaccinated *Tlr4*-deficient mice the number of bacteria, and lung IL-1α and IL-1β expression are higher than in similarly treated wild-type controls, while bronchial LN cell IFN-γ and IL-17 production and splenocyte TNF-α production are lower. Table 1 summarizes our findings. In non-vaccinated but also in vaccinated animals, before challenge no effect of the *Tlr4 *mutation on Ptx-, FHA-, and Prn-IgG levels is seen. After challenge of wP-vaccinated *Tlr4*-deficient mice, the number of bacteria, Prn-IgG level, and lung IL-5 expression are higher compared to wild-type controls, and lung pathology is diminished, while lung IL-1β, TNF-α, IFN-γ, IL-17, and IL-23 expression and bronchial LN cell IL-17 production are lower. After challenge of aP-vaccinated *Tlr4*-deficient mice the number of bacteria, Prn-IgG level, and lung IL-5 expression are higher compared to wild-type controls, while Ptx-IgG level and weight gain are lower. Collectively, these data show an important role for Tlr4 in infection, being bacterial clearance, induction of pro-inflammatory cytokines, and induction of Th1 and Th17 responses. Tlr4 is also important in the challenge response after wP vaccination, being bacterial clearance but also concomitant pathology, induction of pro-inflammatory cytokines, and induction of Th1, Th17, and Th2 responses. Tlr4 plays an important role also in the challenge response after aP vaccination, with a more limited number of parameters affected, being bacterial clearance and induction of the Th2 response. Our data confirm previous data by Higgins et al. [7], who showed an important role of Tlr4 in induction of Th17 cells by infection, in bacterial clearance after wP- and aP-vaccination, and induction of Th1 and Th17 cells by wP but not aP vaccination.

**Table 1:** summary of Tlr4 mutation effects (Tlr4^Lps-d ^vs. wild-type)

	Pre-challenge	Post-challenge		Post-challenge		Post-challenge	
		Non-vaccinated		wP-vaccinated		aP-vaccinated	

Number of bacteria		↑		↑		↑	
Ptx-IgG	=^1^	ND^3^		ND		↓	
FHA-IgG	=^2^	ND		=		=	
Prn-IgG	=^2^	ND		↑		↑	
Weight gain		=		=		↓	
Lung pathology		=		↓		=	
Pro-inflammatory cytokines^4^		↑	↓	↓	↓	=	=
Th1/Th17 cytokines^4^		=	↓	↓	↓	=	=
Th2 cytokines^4^		=	ND	↑	ND	↑	ND

^1^Only aP-vaccinated mice showed detectable Ptx-IgG levels. ^2^Only wP- and aP-vaccinated mice showed detectable FHA-IgG and Prn-IgG levels. ^3^ND, not detected. ^4^First symbol indicates mRNA expression; second symbol indicates production.

Concerns have been raised with respect to the relative efficacy of aP as compared to wP, and also of vaccines administered simultaneously with aP, such as diphtheria, tetanus, polio, and *Haemophilus influenzae *b (Hib) vaccines [9]. A cause for the limited efficacy of aP may be that, in contrast to wP, they do not contain LPS. In mice Th17 [10] and Th1 cells are induced by wP vaccination, but not (or to a much lesser extent) by aP vaccination, and the induction of these Th17 and Th1 cells is Tlr4-dependent [7]. Importantly, Th17 and Th1 cells are critical for clearing a *B. pertussis *challenge [7]. These findings show the importance of Tlr4 (and hence, also its agonists such as LPS) in generating immune responses induced by pertussis vaccines, at least in mice. The efficacy of vaccines that do not contain LPS, such as viral vaccines and bacterial subunit vaccines can be improved by supplementation with Tlr4 agonists that show no or little endotoxin activity. We have shown that supplementing the bacterial subunit vaccine aP with the Tlr4 agonist monophosphoryl lipid A improved its efficacy [8].

On day 3 after infection vaccinated mice had a lower number of bacteria in their lungs than adjuvant controls. This may have affected the cytokine levels measured. Therefore, we plan to study cytokine responses earlier in infection to determine their effects on bacterial clearance.

Our data show that Tlr4 affects the humoral response to vaccination only after challenge. This suggests a Tlr4-dependent effect of bacterial challenge on the humoral response, and that Tlr4 plays a more important role in the challenge phase than in the vaccination phase. Transfer studies from vaccinated *Tlr4*-deficient mice to naive *Tlr4*-sufficient animals and subsequent challenge, or studies using conditional *Tlr4*-knockout mice should settle this issue.

Our failure to detect Ptx-IgG in wP-vaccinated animals is in line with observations in both humans [11] and mice [12,13] that showed aP vaccines to induce a much higher Ptx-IgG level than wP vaccines. In rather contrast to a previous report where Prn-IgG could be detected in convalescent serum from *B. parapertussis *but not *B. pertussis *infected mice [14], we did observe Prn-IgG in vaccinated mice. The lower FHA- and Prn-IgG levels after challenge may be explained by Ptx-mediated suppression of serum antibody levels after infection [15] or by binding of the antibodies by the bacterial challenge.

We have identified an association of the minor allele of rs2770150 in TLR4 with a lower Ptx-IgG level in wP-vaccinated children [6]. As we did not detect Ptx-IgG after wP vaccination of mice, we cannot compare the data obtained in humans to those in mice.

Here we show that Tlr4 plays an important role in lung pathology induced by wP vaccination and challenge. Until recently, it has been assumed that tissue damage is mediated by Th1 cells [16]. We have previously shown, however, that Th1 cells are not involved in lung pathology induced by wP vaccination and challenge, as T-bet KO mice that lack Th1 cells showed similar pathology compared to wild-type controls [5], suggesting that a different subset is involved. Possibly, Th17 cells may be the subset responsible for this pathology, as these cells are important in mediating tissue damage [16,17] and are induced by wP vaccination in a Tlr4-dependent way [7]. Th17 cells are induced in the presence of IL-6, while regulatory T-cells are induced in the absence of this cytokine (in an otherwise similar cytokine milieu) [17]. Possibly, induction of IL-6 by wP vaccination [8] may play a role in the generation of Th17 cells. Whether indeed Th17 cells are responsible for lung pathology after wP vaccination and challenge awaits further study.

Our finding of infection-induced IL-17 production is in line with the observation of IL-23 production by human monocyte-derived DC after *B. pertussis *infection [18]. Since IL-23 is required for amplifying and/or stabilizing Th17 cells [10], both findings suggest Th17 induction by *B. pertussis *infection.

It is intriguing but also complicating that not only LPS but also Ptx may play a role in the Tlr4-dependent induction of Th17 cells. Ptx has Tlr4-dependent adjuvant activity [19,20] and is able to induce Th17 cells [21]. It is likely, but not formally proven, that this latter effect is Tlr4-dependent (X. Chen, personal communication). Vaccination with wP that is derived from Ptx-deleted *B. pertussis *may clarify this issue.

We show that production of IFN-γ and IL-17 by bronchial LN cells from non-vaccinated *Tlr4*-deficient mice is lower than from wild-type controls, in line with previous observations for IFN-γ [2] (IL-17 was not measured in that study). This suggests that not only wP vaccination but also *B. pertussis *infection induces Th1 and Th17 cells in a Tlr4-dependent way.

IL-10 production by *B. pertussis*-stimulated splenocytes is lower when these cells are obtained from *Tlr4*-deficient mice than from wild-type controls. Challenge-induced pathology is similar between adjuvant-treated *Tlr4*-deficient mice and similarly treated wild-type controls, and lower in wP-vaccinated *Tlr4*-deficient mice than in their wild-type controls. Together, these data suggest that in our model IL-10 is not involved in limiting pathology, in contrast to a study by others [2]. Our observations were made at day 3 after challenge, however, whereas the other study [2] made these observations at day 14–21.

A recent paper has shown that the number of bacteria in the lungs of *B. pertussis *infected TNF-α deficient mice was higher than in similarly treated wild-type controls [22]. In our study, *B. pertussis*-stimulated splenocytes from infected *Tlr4*-deficient mice produce less TNF-α than cells from similarly treated wild-type controls. The lower systemic TNF-α response observed in our study may contribute to the higher number of bacteria in the lungs of *Tlr4*-deficient mice. It has to be noted, however, that the differences in bacterial numbers between TNF-α deficient mice and their controls were not seen until day 10 after infection [22].

Two signal transduction pathways downstream of Tlr4 have been identified, the MyD88-dependent pathway and the MyD88-independent (TRIF) pathway. The former pathway involves the adaptor proteins TIRAP and MyD88, the latter one TRIF and TRAM [23]. The bacterial species from which LPS originates, determines the pathway(s) activated [24]. Since wP is derived from *B. pertussis*, they harbor the same LPS suggesting activation of the same pathway. wP, however, consists of glutaraldehyde-fixed bacteria, and its LPS may thus activate a different pathway than LPS from live *B. pertussis *[25]. To study possible differences in pathway activation, *TRIF*-deficient (C57BL/6J-Ticam1^Lps2^/J [26]) and wild-type controls (C57BL/6J) were compared in our model. *TRIF*-deficient mice are readily available and are not deficient in other pathways besides TRIF/TRAM, while MyD88 KO mice are not only deficient in MyD88/TIRAP but also in the IL-1R and IL-18R pathways [27]. Three days after infection, wP- and aP-vaccinated mice showed a ~500-fold lower colonization than non-vaccinated controls, and no differences in colonization were seen between TRIF-deficient and wild-type mice (data not shown). This shows that the TRIF pathway is not involved in clearance of a *B. pertussis *infection, nor is it involved in wP or aP vaccine-induced clearance.

TRIF-deficient mice are strongly impaired in LPS-induced CD40, CD80, CD86, and MHC class II upregulation, as well as type I IFN production in macrophages *in vitro*, and CD40, CD80, and CD86 upregulation in CD11^+ ^DC *in vivo*. Also, the CD4^+ ^and CD8^+ ^T-cell response is abolished when LPS is used as adjuvant [28]. Importantly, type I interferons promote memory T-cell proliferation [29]. Thus, the lack of an effect of *TRIF*-deficiency suggests that clearance of a *B. pertussis *infection, as well as wP- and aP-induced immunity are also functional under conditions of severely reduced costimulation and (memory) T-cell responses. This lack of an effect of *TRIF*-deficiency also suggests involvement of the MyD88-dependent pathway. We set out to study this pathway, but breeding problems with MyD88 KO mice prohibited these experiments. Still, a role for MyD88 is likely from a study by Togbe et al. [30] who showed a TIRAP- and MyD88-dependent TRIF-independent response to LPS in the lungs, including Th1 cytokine production and neutrophil influx. Both parameters are characteristic of *B. pertussis *challenge, as well as of wP but not aP vaccination and similar challenge [12,31].

## Conclusion

**Figure 7:**
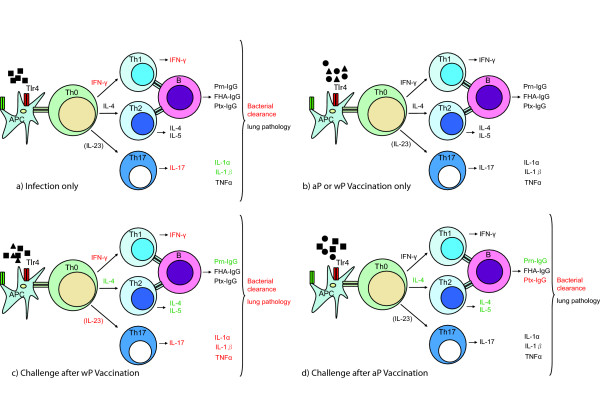
**Effects of *Tlr4 *mutation in vaccine-induced immunity**. Parameters that show a lower response in *Tlr4*-deficient mice compared to wild-type animals are represented in red, while the ones that show a higher response are represented in green. Parameters that are not affected or have not been determined are represented in black. Triangles represent wP, circles aP, and squares *B. pertussis*. IL-23 is put between brackets as this cytokine is able to maintain, rather than generate Th17 cells.

We have shown that Tlr4 does not affect the humoral response to vaccination. Tlr4 is important in (i) natural defense to *B. pertussis *infection, and also in wP and aP vaccination-induced clearance, (ii) challenge-induced lung pathology after wP vaccination, (iii) induction of Th1 and Th17 responses by *B. pertussis *infection and also by wP vaccination and challenge, and (iv) reduced Th2 response after wP and aP vaccination and challenge. A summary of our findings is presented in Figure 7.

## Methods

### Animals

Female mice were used at 6–8 weeks of age. Tlr4^Lps-d ^C3H/HeJ [32] and wild-type control C3H/HeOuJ mice were obtained from Jackson (Bar Harbor, ME). The diet consisted of ground standard laboratory chow (RMH-B, Hope Farms, Woerden, the Netherlands). Food and water were given ad libitum. All animal experiments were performed according to national and international guidelines.

### Vaccines

The wP vaccine is a whole-cell pertussis vaccine, combined with vaccines against diphtheria, tetanus, and poliomyelitis, and produced by the Netherlands Vaccine Institute, Bilthoven, the Netherlands. One human dose (HD) contains 1.6 × 10^10 ^CFU of two *B. pertussis *strains (strains 509 and 134; 8 opacity units/strain) in 1 ml saline, and is adjuvated with 1.5 mg aluminum phosphate.

The aP vaccine is 3-component acellular pertussis vaccine, combined with vaccines against diphtheria and tetanus, and produced by GlaxoSmithKline, Rixensart, Belgium. One HD contains 25 μg formaldehyde- and glutaraldehyde-detoxified pertussis toxin, 25 μg filamentous hemagglutinin, and 8 μg pertactin in 0.5 ml saline, and is adjuvated with aluminum hydroxide (< 0.625 mg Al per HD).

### Vaccination

One HD wP or aP was diluted with 0.5% Al(OH)_3 _gel (Serva, Heidelberg, Germany) to a final volume of 2.5 ml. Mice (6 per group) received a subcutaneous injection with 0.5 ml of 1/5 HD wP, 1/5 HD aP, or adjuvant alone, 28 and 14 days before infection [5,13]. Note that the ratio between adjuvant and bacteria (in case of wP) or between adjuvant and proteins (in case of aP) was considerably higher in the murine vaccine doses than in the original human vaccine doses.

### Infection of mice and autopsy

The mice were anaesthetized with isoflurane. Two h before infection, 3 drops of blood were collected from the orbital plexus. A single drop of 40-μl inoculum containing 2 × 10^7 ^*B. pertussis *cells (Tohama strain B213) was carefully placed on the top of the nose and allowed to be inhaled [33].

Mice were sacrificed 3 or 7 days after challenge, except in case of lung pathology in which case the mice were sacrificed 3 days after infection only. They were anaesthetized with ketamine, rompun, and atropine, and blood was collected from the orbital plexus. Perfusion of the right ventricle was performed with 2 ml PBS supplemented with 3.5% heat-inactivated Fetal Calf Serum (FCS; PAA, Linz, Austria). The lungs were excised and used to obtain bronchial lymph nodes (LN) and lung lobes. The left lung lobes were formalin-fixed for histological examination or collected in RNA-later (Qiagen, Venlo, the Netherlands) for RNA extraction, while the right lung lobes were used for enumeration of bacteria.

### Lung lobes, CFU determination, and histological examination

A ligature was made around the right bronchus after which the right lobes were removed for enumeration of bacteria. The lobes were homogenized in 900 μl of Verwey medium using a tissue homogenizer (Pro-200, ProScientific, Monroe, CT) at maximum speed for 10 s. The homogenates were diluted in Verwey medium 10- and 100-fold for the immunized mice, and 1000-fold for the control mice. Hundred-μl aliquots of the dilutions were plated on BG plates supplemented with streptomycin and incubated at 35°C for 5 days.

The remaining left lung lobes were fixed intratracheally using 4% formalin for 24 h. After overnight dehydration, they were embedded in paraffin. Five-μm sections were cut and stained with haematoxylin/eosin. Histological lesions were semi-quantitatively scored as absent (0), minimal (1), slight (2), moderate (3), strong (4), or severe (5), respectively. This score incorporates the frequency as well as the severity of the lesions.

### Ptx-, FHA-, and Prn-specific IgG

Pertussis toxin (Ptx) and filamentous hemagglutinin (FHA) were obtained from Kaketsuken, Kumamoto, Japan. Pertactin (Prn1) was prepared as described previously [34]. Immulon 2HB plates (Thermo Scientific, Waltham, MA) were coated with 2 μg/ml Ptx, 2 μg/ml FHA, or 5 μg/ml Prn1, in PBS, incubated overnight, and washed. After addition of a dilution series of control and test sera, the plates were incubated for 1 h and washed. After addition of detection antibody (1:5,000 anti-mouse IgG-horseradish peroxidase (HRP); Southern Biotechnology Associates, Birmingham, AL) in PBS, 0.1% Tween-80, 0.5% Protifar (Nutricia, Zoetermeer, the Netherlands), the plates were incubated for 1 h and washed. To detect HRP, the plates were washed and incubated for 10 min in 10% sodium acetate, 1% tetramethylbenzidine (Sigma, Axel, the Netherlands), and 0.01% H_2_O_2_. 2M H_2_SO_4 _was added to stop color development and the plates were read at 450 nm. Washing steps were 5 times with PBS-0.03% Tween-80. Incubations were at room temperature.

### Cell culture

The culture medium used was RPMI-1640 (Gibco, Grand Island, NY) supplemented with 10% FCS, 100 μg/ml streptomycin, and 100 IU/ml penicillin. Cell suspensions were made by pressing the LN or spleens through a cell strainer (Falcon, Franklin Lakes, NJ). Cells were counted using a Coulter Counter (Coulter Electronics, Luton, UK). LN cell suspensions were cultured at 10^6 ^cells per ml culture medium with 5 μg/ml Concanavalin A (Con A; MP Biomedicals, Irvine, CA) in flat-bottom 12-well culture plates (Costar, Cambridge, MA) at 37°C in a humidified atmosphere containing 5% CO_2 _for 24 h. Spleen cell suspensions were cultured at 10^6 ^cells per ml culture medium with 5 μg/ml Con A or *B. pertussis *(10^5 ^heat-inactivated bacteria per well) in 96-well tissue culture plates (Nunc) at 37°C in a humidified atmosphere containing 5% CO_2 _for 72 h. Bacteria were heat-inactivated at 56°C during 30 min.

### Cytokine expression

Cytokine mRNA expression was measured using Taqman gene expression assays (Applied Biosystems, Foster City, CA) as described previously [4,5]. Briefly, RNA was extracted with an RNeasy kit (Qiagen). Copy DNA was generated using the High Capacity cDNA archive kit containing random hexamer primers (Applied Biosystems). Messenger RNA expression was measured on a 7500 Fast Real-Time PCR System (Applied Biosystems). We used the assay on demand (Applied Biosystems) for IL-1α (Mm99999060), IL-1β (Mm01336189), IL-4 (Mm00445259), IL-5 (Mm00439646), IL-17 (Mm00439618), IL-23 (Mm00518984), IFN-γ (Mm00801778), and TNF-α (Mm00443258). For the reference gene, hypoxanthine phosphoribosyl transferase (HPRT), the assay was designed using the primer express program (Applied Biosystems) resulting in probe CAGTCCTGTCCATAATCA, forward primer GCCGAGGATTTGGAAAAAGTGTTTA, and reverse primer TTCATGACATCTCGAGCAAGTCTTT. The relative concentration of the various mRNA's was determined by the comparative threshold cycle method (ddCt) [35-37].

The fold change in mRNA expression is relative to lung tissue of untreated C57BL/6J mice. This third-party control strain does not favor either of the two strains of mice tested (the C3H/HeJ or the C3H/HeOuJ strain).

### Cytokine measurements

An 8-plex panel containing beads for mouse IL-1α, IL-4, IL-5, IL-10, IL-13, IL-17, IFN-γ, and TNF-α (Bio-Rad, Hercules, CA) was used. After incubation and washing steps (see [5] for details) the beads were measured on a Bio-Plex (Bio-Rad).

### Statistics

One-way analysis of variance (ANOVA), followed by the Bonferroni post-hoc test was performed (SPSS, Chicago, IL). Changes in body weight were analyzed by paired sample testing (SPSS). Histological data were analyzed using the non-parametric Mann-Whitney U test (SPSS).

## Authors' contributions

SB carried out the infection and real-time PCR experiments and helped to draft the manuscript. RMS developed and carried out the serology. ERG carried out the cell culture and immunoassays. JAMAD evaluated the lung pathology. FRM participated in the study design and coordination. TGK conceived the study, participated in the study design and coordination, and helped to draft the manuscript. RJV conceived the study, participated in the study design and coordination, and wrote the manuscript. All authors read and approved the final manuscript.
